# Ebola Cases and Health System Demand in Liberia

**DOI:** 10.1371/journal.pbio.1002056

**Published:** 2015-01-13

**Authors:** John M. Drake, RajReni B. Kaul, Laura W. Alexander, Suzanne M. O’Regan, Andrew M. Kramer, J. Tomlin Pulliam, Matthew J. Ferrari, Andrew W. Park

**Affiliations:** 1 Odum School of Ecology, University of Georgia, Athens, Georgia, United States of America; 2 Department of Biology, Pennsylvania State University, State College, Pennsylvania, United States of America; 3 College of Veterinary Medicine, University of Georgia, Athens, Georgia, United States of America; Imperial College London, UNITED KINGDOM

## Abstract

In 2014, a major epidemic of human Ebola virus disease emerged in West Africa, where human-to-human transmission has now been sustained for greater than 12 months. In the summer of 2014, there was great uncertainty about the answers to several key policy questions concerning the path to containment. What is the relative importance of nosocomial transmission compared with community-acquired infection? How much must hospital capacity increase to provide care for the anticipated patient burden? To which interventions will Ebola transmission be most responsive? What must be done to achieve containment? In recent years, epidemic models have been used to guide public health interventions. But, model-based policy relies on high quality causal understanding of transmission, including the availability of appropriate dynamic transmission models and reliable reporting about the sequence of case incidence for model fitting, which were lacking for this epidemic. To investigate the range of potential transmission scenarios, we developed a multi-type branching process model that incorporates key heterogeneities and time-varying parameters to reflect changing human behavior and deliberate interventions in Liberia. Ensembles of this model were evaluated at a set of parameters that were both epidemiologically plausible and capable of reproducing the observed trajectory. Results of this model suggested that epidemic outcome would depend on both hospital capacity and individual behavior. Simulations suggested that if hospital capacity was not increased, then transmission might outpace the rate of isolation and the ability to provide care for the ill, infectious, and dying. Similarly, the model suggested that containment would require individuals to adopt behaviors that increase the rates of case identification and isolation and secure burial of the deceased. As of mid-October, it was unclear that this epidemic would be contained even by 99% hospitalization at the planned hospital capacity. A new version of the model, updated to reflect information collected during October and November 2014, predicts a significantly more constrained set of possible futures. This model suggests that epidemic outcome still depends very heavily on individual behavior. Particularly, if future patient hospitalization rates return to background levels (estimated to be around 70%), then transmission is predicted to remain just below the critical point around *R*_eff_ = 1. At the higher hospitalization rate of 85%, this model predicts near complete elimination in March to June, 2015.

## Abstract 2

The authors develop a multi-type branching process model of the 2014 Liberian Ebola outbreak that incorporates the impacts of changes in behavior on potential transmission scenarios, thereby informing the path to containment of the epidemic.

## Author Summary

There is considerable uncertainty regarding the steps needed to contain the ongoing Ebola crisis in West Africa, the timeline required to achieve control, and the projected burden of mortality. To address these issues, we develop a branching process model for Ebola transmission that focuses on offspring distributions (i.e., the numbers of new infections caused by each case). We use the model to assess the likely progression of Ebola in Liberia. The model assesses the feedback between new cases and hospital demand under a range of plausible intervention scenarios, particularly ramping-up of treatment facilities over time and increasing the number of individuals seeking hospital treatment through outreach and education. Transmission scenarios—to health care workers in hospitals, to caregivers in the community, to hospital visitors, and to individuals preparing bodies for funerals—are described by distinct offspring distributions based on available data. Results suggest that the outcome of the epidemic depends on both hospital capacity and individual behavior. Additionally, the model highlights the conditions under which transmission might have outpaced hospital capacity, and projects possible epidemic trajectories into 2015.

## Introduction

The 2014 epidemic of Ebola virus in West Africa is an emerging public health and humanitarian crisis of epic dimensions [1]. This epidemic originated in an outbreak in Guéckédou, Guinea in December 2013. The Ministry of Health of Guinea and Médecins Sans Frontières (MSF) were alerted to clusters of an unknown disease with fever/vomiting/diarrhea and a high fatality rate on 10 and 12 March 2014, respectively [2]. Through human-to-human transmission, the virus subsequently spread to Liberia (29 March [3]), Sierra Leone (25 May [4]), Nigeria (22 July [5]), Senegal (29 August [6]), the United States (30 September [7]), and Mali (30 September [8]). On 8 August 2014, WHO determined the epidemic to be a “Public Health Emergency of International Concern.” This declaration obligated 194 signatory nations to participate in disease prevention, surveillance, control, response, and reporting [9]. On 6 October, the first transmission outside of Africa was documented in Spain [10]. As of 14 December, 18,603 persons were reported (but not confirmed) to have been infected with a fatality rate for those cases with known clinical outcome around 70% [1]. Due to widespread under-reporting, the true number of cases is widely believed to be considerably higher.

Ongoing international support has included the shipment of large quantities of personal protective equipment, diagnostic laboratory apparatus, and materiel such as vehicles; provision of medical and logistical advisors from MSF, the US Centers for Disease Control & Prevention, and WHO, among others; and the construction of new treatment facilities [11]. A range of further clinical interventions, health policies, and aid are under consideration and at various stages of mobilization. Whether these are sufficient to achieve containment and/or what further actions might extend their reach remain unknown.

Epidemic modeling provides a means for structured reasoning about such complex dynamical conditions, both with respect to the information contained in this epidemic’s history to date and prospective opportunities for intervention. While several models of the 2014 West Africa Ebola epidemic have been published, the majority of these are primarily aimed at estimating the basic reproduction number (*R*_0_), a summary statistic that may be tremendously informative about the potential rate of spread and the magnitude of vaccination required to achieve herd immunity [12–14]. Knowing *R*_0_ is less useful where human behaviors—including both public health interventions [15] and avoidance or denial in the community [16]—cause the epidemic to take a more irregular path [17]. Two models that incorporate more detail have been published. A paper by the WHO Response Team [1] proposes a renewal equation for the evolution of the epidemic through time, parameterized with case reports collected by MSF. But this model, which focuses on the time course of disease and conditions for transmission, does not account for the role of transmission setting. The model of Meltzer and colleagues [18] is more tactical, but provides little analytical insight.

Here, we report on a model of intermediate complexity. Our goal was to produce a model that could be used to guide policy recommendations. A supporting objective was to perform analysis of a range of scenarios to identify how actions taken in the present may influence short and medium term prospects for containment. The model comprises separate probability distributions for the number of secondary cases arising among health care workers (HCWs) infected in hospitals, non-HCWs infected by hospitalized patients, non-HCWs infected during non-hospital nursing care, and non-HCW infected through burial practices. Infected individuals may be treated in the hospital or in the home. Hospital treatment is assumed to result in reduced transmission but is limited to a fixed number of available hospital beds. Cases in excess of hospital capacity are assumed to be treated in the home. Cases seeking hospitalization (whether capacity allows admission or not) are scored as a report, separating the total number of cases (which is unknown) from the number of cases reported. In contrast to the models in [1] and [18], this model allows for changing human behavior and epidemic interventions through time-varying rates of hospitalization, exposure of HCWs, and secure burial [19]. We use the theory of branching processes to derive an expression for the mean number of secondary infections.

## Methods

### Data

**Figure 1:**
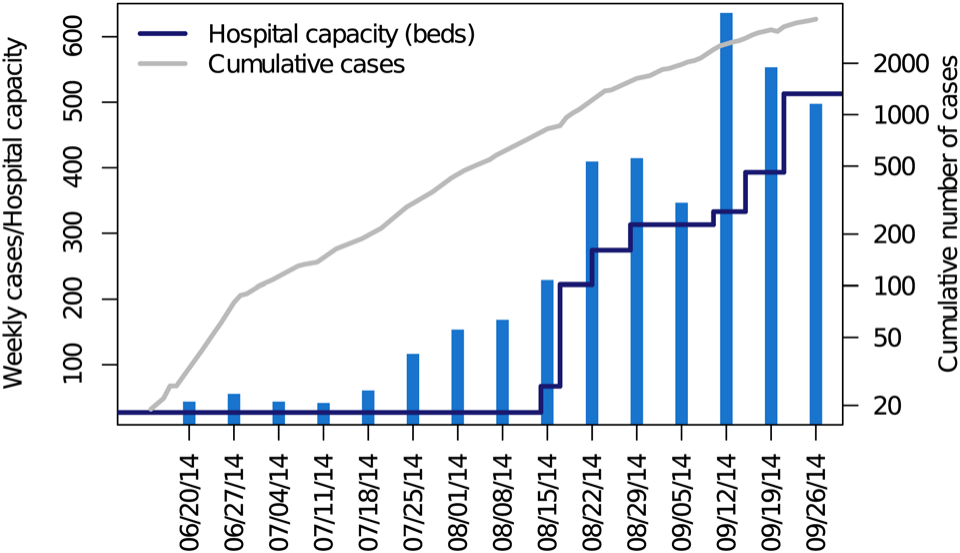
Weekly number of suspected, probable, and confirmed cases of Ebola virus in Liberia in the seven days terminating with each date (blue bars) and daily cumulative reports (gray line). Hospital capacity in Ebola treatment units (total number of beds in country, dark blue line) was compiled from non-governmental organizations, media, and government sources. Beginning in September 2014, the WHO situation reports were considered to be unreliable with respect to the timing of case reports and the epidemic curve is discontinued. The underlying data and code to generate this figure may be obtained by running the file “ebola-forecasting-main-text.R” deposited in the Dryad repository: http://doi.org/10.5061/dryad.17m5q; data from WHO situation reports.

Data were obtained from situation reports issued by WHO and the Liberia Ministry of Health (Fig. 1). All situation reports were pulled from the Liberia Ministry of Health or United Nations Office for the Coordination of Humanitarian Affairs (UN-OCHA) websites (http://reliefweb.int and http://humanitarianresponse.info). When values had to be interpolated, data from WHO outbreak reports were used. For provenance and reproducibility, we digitally entered our own data (data deposited in the Dryad repository: http://doi.org/10.5061/dryad.17m5q [20]). Reported cases were scored as the sum of suspected, probable, and confirmed cases.

### A Branching Process Model

**Figure 2:**
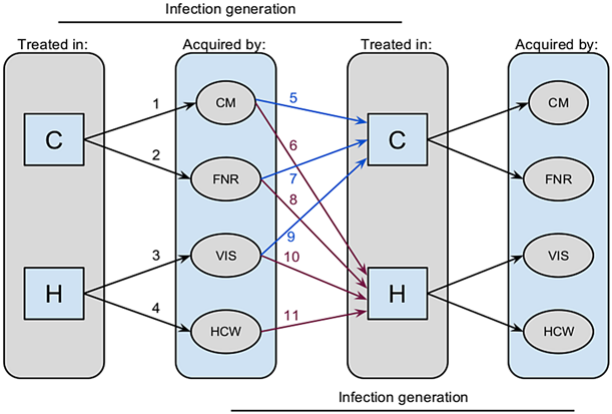
Structure of a model for human-to-human transmission of Ebola virus. Flow of transmission is depicted through two generations of infection in a multi-type branching process model of Ebola virus transmission. Grey panels show that infected persons may be treated in either the community (C, blue paths) or hospital (H, purple paths). Community-treated patients may give rise to secondary infections in community members through nursing care (CM) or in the process of body preparation and burial (FNR). Hospital-treated patients may give rise to secondary infections in HCWs or visitors (VIS). Infected persons may either be treated in the community or in the hospital at rates that depend on the conditions under which the infection was acquired.

**Model**. We developed a discrete time, stochastic process model for Ebola transmission. The model considers the context in which transmission occurs and who is infected as a result. This framework allows a minimal set of subpopulation differences to be articulated that nonetheless reflect the major epidemiological properties of Ebola transmission, including hospital treatment versus community care, transmission at funerals, and scenario-dependent transmission risk differences during care-giving. The model comprises separate probability distributions for the number of secondary cases arising from (i) HCWs infected in hospitals, (ii) non-HCWs infected by hospitalized patients, (iii) non-HCWs infected during non-hospital nursing care, and (iv) non-HCW infected through burial practices. Infected individuals are considered to be treated either in the hospital or in the home (Fig. 2).

Specifically, our model supposes that transmission is composed of five processes that result in 11 state transitions (Fig. 2). In the following description, numbers in parentheses correspond to labels in Fig. 2.

- Persons treated in the community give rise to a Poisson distributed number of secondary infections among community members at rate *λ* = *Nq*, where *N* is the number of contacts and *q* is the per contact probability of transmission. To accommodate heterogeneity in transmission, *λ* may be taken to be a random variable, in which case the number of secondary infections is negative binomially distributed with an additional parameter, *θ*, regulating dispersion (1).
- Persons treated in the hospital give rise to a Poisson distributed number of secondary infections among HCWs at rate *λβα*, where *β* is a multiplier for the additional contacts acquired through hospitalization and *α* is a multiplier for the effect of infection control interventions (2).
- Persons treated in the hospital may infect a Poisson distributed number of visitors at rate *λ_h_* (3). It is assumed that all deceased hospitalized patients are given a secure burial.
- Persons treated in the community recover or are given a secure burial at rate *g* (and therefore do not give rise to any further secondary infections) and non-secure burial at rate 1 − *g*, giving rise to a Poisson distributed number of secondary infections at rate *ϕ* (4).
- Persons acquiring infection in the community (classes CM, VIS, and FNR) are hospitalized with probability *h* (6, 9, 11) or remain in the community with probability 1 − *h* (5, 8, 10).
- Infected HCWs are all assumed to be hospitalized (7).

These processes constitute a multi-type branching process composed of mixtures and convolutions of the core probability distributions (Box 1). Branching process models allow for very flexible specification of the distribution of secondary cases. Our branching process does not account for the depletion of susceptibles at the population level, however, and is therefore appropriate during the exponential phase of epidemic spread and/or where spread is controlled through human intervention rather than self-limitation. We believe these assumptions are broadly consistent with the currently prevailing conditions in West Africa.

**Box 1. Branching Process Model**

Our model is made from mixtures and convolutions of the base distributions of secondary cases associated with community-based nursing, hospital care, and body preparation and burial. A mixture distribution describes the probability of random variables drawn from one of two or more component distributions. A convolution distribution describes the probability of random variables obtained by summing two or more other random variables. For instance, in our model, the distribution of secondary cases for persons who are treated in the community is a mixture of the secondary cases generated by those who recover or are provided a safe burial (which occurs with probability 0≤*g*(*t*)≤1) and those who die and are not provided a safe burial (which occurs with probability 0≤1−*g*(*t*)≤1). Secondary cases due to individuals who recover or receive safe burial derive exclusively from community-based transmission and are distributed as a negative binomial distribution with intensity parameter *λ* = *Nq* and dispersion *θ*. Secondary infections due to deceased patients who are not provided a safe burial is the sum of those due to community-based transmission and those due to funerary transmission. By assumption, funerary transmission gives rise to a Poisson distributed number of secondary infections with rate parameter *ϕ*. The convolution of a negative binomial distribution and a Poisson distribution is a Delaporte distribution. Thus, the distribution of total number of secondary infections from persons treated in the community is the mixture of a negative binomial distribution and a Delaporte distribution (arrows 1 and 2 in Fig. 2). Secondary cases generated by patients treated in the hospital is also a convolution of the infections of HCWs and visitors (arrows 3 and 4 in Fig. 2). Since the convolution of two Poisson distributions is a Poisson distribution, this distribution of secondary cases is Poisson with rate parameter (*λβα+λ_h_*). The number of secondary cases in each infection generation is a mixture (with mixing parameter *h*) of community-acquired, funerary, and hospital-visitor-acquired cases that are treated in the community (arrows 5, 7, and 9 in Fig. 2) and those that are treated in a hospital (arrows 6, 8, 10, and 11 in Fig. 2). The different forecasting scenarios we analyzed reflect assumptions about how the availability of hospital beds and hospitalization rates affect this mixture. These considerations directly lead to the mean matrix of secondary infections by type (equation 1 in S1 Text) and the analytic expression for *R_eff_* (equations 2 and 3 in S1 Text).

**Hospital capacity.** In simulations, hospital treatment was assumed to result in reduced transmission, limited by the number of available hospital beds. Patients seeking hospitalization in excess of hospital capacity were assumed to be returned to the home for treatment. Only patients seeking hospitalization (whether capacity allowed admission or not) were scored as a report, separating the total number of cases (which in reality is unknown) from the number of cases reported.

### Parameterization

To parameterize this model, we were initially guided by reports on the outbreaks of Ebola virus in Kikwit (Democratic Republic of Congo) in 1995 [21–24] and Gulu (Uganda) in 2000–2001 [25–27]. The values obtained in this section were used as a starting point for a more systematic analysis, as described in the section “Plausible Parameter Sets.”

**Transmission (*N*, *q, θ*) and the effectiveness of infection control (*α*)**. The attack rate in Kikwit was 9% among hospital workers [28] and 16% among family members [22]. The ratio of exposures to index cases in households was *N* = 173/27 = 6.4 for 27 different families. Assuming exposure was only within the family (so each secondary case had only one exposure), we have *q* = 0.16 (risk of transmission per contact). At Kikwit General Hospital, 37 of 429 workers met the case definition for Ebola virus disease. A reported three cases occurred after the use of barrier nursing. If we assume that these three were all in Kikwit General Hospital, then 34 HCWs were infected prior to infection control. A total of 110 out of 138 other hospital workers reported direct contact with an Ebola patient. Extrapolating to the 392 HCWs who weren’t infected, we estimate the number of workers with direct contact to be 110/138×392+34≈ 346 yielding an attack rate of 9.8%. Of course, hospital workers experience greater exposure than persons providing care in the community. Among 48 uninfected persons with direct contact jobs at Kikwit General Hospital there were a total of 151 patient contacts (3.15 contacts per worker). If this were representative, then we would have the relation 1−(1−*qα*)^3.15^ = 0.098, yielding *α =* 0.20 prior to the implementation of barrier nursing and other infection control measures. Following barrier nursing, three out of 110/138×392+3≈ 315 HCWs were infected, yielding an attack rate of 0.95%. Using the relation 1−(1−*qα*)^3.15^ = 0.0095 we obtain *α* = 0.019 after the implementation of barrier nursing and other infection control measures.

**Hospital contact multiplier (*β*).** The parameter *β* relates the number of contacts in a health facility to those in a household and is expressed as a multiplier of *N*. This value is chosen based on intuition and narrative reports. In general, we consider values in the range 2<*β<*5 to be reasonable.

**Funeral transmission (*ϕ*).** Legrand and colleagues [29] assumed that mean duration of death to burial was 2 days and estimated transmission rates of 7.66 per week (Kikwit) and 0.46 per week (Gulu). Translating into average number of infections, the number of secondary cases through funeral are estimated to be 2.18 and 0.13, respectively, assuming *S*/*N*≈1, where *S* is the number of susceptible individuals in the population and *N* is the total population size. A value of 0<*ϕ<*3 is consistent with the routine finding that preparation of the body constitutes a substantial risk factor and that this duty is performed by a relatively small number of people. We note that this is not consistent with anecdotal reports of large numbers of persons being infected at a funeral. We consider those events most likely to be exceptional. Parameter values of this “core model” are reported in Table 1.

**Table 1:** Parameter values of the basic branching process model for Ebola transmission.

**Variable**	**Value**
Household contacts (*N*)	6.4
Transmission probability (*q*)	0.16
Overdispersion (*θ*)	1
Hospital contact multiplier (*β*)	4
Effectiveness of infection control (*α*)	0.019
Average number of secondary community cases from hospitalized patients (*λ_h_*)	0.3
Average number of secondary cases from a funeral (*ϕ*)	2.18

**Treatment facilities.** From a range of reports, we compiled a time series of the operational Ebola treatment units (ETUs) along with estimates of their capacity, recorded as the number of patient beds available (Fig. 1). Importantly, many ETUs were regularly reported to be operating above capacity, typically by around a factor of two [30–32]. Additionally, the average hospital stay is around 6.5 days [1], considerably shorter than the 15-day infection generation. Therefore, throughout our analysis, we estimate the number of patients potentially served by an ETU within an infection interval using the formula

s(t)=2b(t)τ/σ. (1)

where *t* marks time in infection generations, *b*(*t*) is hospital capacity in terms of the number of beds, *τ* = 15 is infection generation time, and *σ* = 6.5 is the average duration of hospitalization.

**Secure burial rate.** Non-secure burial (including body preparation and funeral ceremonies) is one of the key occasions for Ebola virus transmission. The Liberia Ministry of Health and international partners have therefore sought to reduce this mode of transmission through public education about the risk of exposure from deceased Ebola patients and the mobilization of body retrieval and burial teams. There has almost certainly been a substantial reduction in transmission due to increased frequency of secure burial. For example, even during the interval from 4 July to 2 September (prior to the downturn [33]), the cumulative reported number of cases shows a negative curvature on a logarithmic scale (grey line in Fig. 1). We therefore modeled *g* (a rate that is the sum of the recovery rate and secure burial rate) using the time-dependent function

$$g\left(t\right)=\gamma_{1}\left(\text{1}-\text{1}/\left(\left(t-\text{7}\right)\gamma_{\text{2}}\right)\right)+\mu .$$ (2)

where *t* is measured in terms of infection generations and *μ* = 0.3 is one minus the case fatality rate, *γ*_1_<0.7 is the maximal secure burial rate, and *γ*_2_ governs the speed at which safe burials increase. This function allows for the secure burial rate to increase beginning around 4 July, starts at a positive minimum due to natural recovery, and asymptotically approaches a maximum at *γ*_1_+*μ*, since we suppose that secure burial and recovery cannot go to 100%.

**Initial conditions.** According to our data, there were 27 beds in ETUs in Liberia on 4 July. Based on the reported cumulative case count, there were approximately 108–30 = 78 active reported cases at this time. Using equation (1), we estimated that 54 of the reported cases were under hospital care. Further assuming under-reporting by a factor of 2.5 [18, 34], we estimated that there were a total of 195 cases for 195–78 = 117 unreported cases at this time. Together, these calculations imply that 54 persons were treated in hospitals and 141 persons were treated in the community.

**Other parameters.** In general, we treat the hospitalization rate (*h*), secure burial rate (*γ*_1_ and *γ*_2_), funeral transmission *ϕ*, and overdispersion (*θ*) as tuning parameters. The time scale of this model is defined with respect to infection generations. To calibrate to calendar time, we assumed a serial interval of 15 days [1]. To calculate hospital capacity, we assumed an average hospital stay of 6.5 days [1].

**Plausible parameter sets.** Guided by these crude parameter estimates, we then tuned our model to data from the 2014 Ebola outbreak in Liberia. There were two waves of transmission in 2014 in Liberia. The first wave occurred in March and April, comprised a total of eight reported cases, and may have gone extinct in mid-May. The second wave began in late May and was the origin of the vast majority of cases. However, reported cases between the end of the first wave and around 4 July were irregular, whereas after 4 July there was a dramatic and sustained increase in the number of cases for many weeks. Around 6 September, the smoothed average number of cases per case (a model-independent estimate of *R_eff_*) began to decline (unpublished data). The WHO Situation Report of 8 October indicates that this decline was probably due to a deterioration in reporting, rather than a true decline in transmission. For these reasons, we focused our fitting on the interval from 4 July 2014 to 2 September 2014. In keeping with the time scale of our model, and to smooth over daily variations in reporting, reported cases were aggregated to 15 day transmission generations (Table 2).

**Table 2:** Reported cases and reported cases among health care workers during five infection generations of the 2014 outbreak of Ebola in West Africa.

**Date**	**Cumulative Cases**	**Cumulative Cases among HCWs**
4 July 2014	122	12
19 July 2014	197	20
3 August 2014	498	64
18 August 2014	972	115
2 September 2014	1,847	153

Data were obtained by aggregating into 15-day intervals the new cases that were irregularly reported by the WHO and Liberia Ministry of Health between endpoints of each interval. Dates in this table represent the last date of an interval.

The parameters *h*, γ_1_, γ_2_, *θ*, *α*, and *ϕ* were first tuned so that the median simulated reports of infection among HCWs in the four infection generations between 4 July and 2 September and the median simulated number of cumulative reports among non-HCWs at the same times were close to the reported values (S1 Fig). We further refined these fits by minimizing squared differences on a logarithmic scale. We then used latin hypercube sampling to explore a parameter space within ±25% of the tuned values. A parameter set was deemed plausible if the reported cumulative number of cases (data) and reported cumulative cases among HCWs (data) were within the range of 500 simulations (model). Further sensitivity analyses were performed and are described in S1 Text.

### Forecasts

To forecast future cases under different scenarios for aid and intervention in the fall of 2014, we projected cases and number of persons seeking hospitalization from 3 September 2014 until 31 December 2014 (120 days) under five scenarios. Details are contained in S1 Text.

By mid-December 2014, it was evident that the effective reproductive ratio at the national level had been reduced to below one—a scenario consistent with, but not guaranteed by the data up until 2 September—significantly reducing the range of epidemic trajectories projected into the future. At this time, we updated the forecasts with a known (rather than conjectural) trajectory for increased hospital capacity including the capacity of referral centers, and by further reducing the set of plausible parameters to contain only those parameterizations consistent with the 5,836 cases observed between 3 September and 1 December. We then simulated future epidemic trajectories into 2015 under this larger base of evidence. The infection generation ending on 1 December comprised 605 reported cases, from which we estimate a total of 1,513 cases. Assuming 72% were admitted for hospitalization (Table 3), we initialized these simulations with 1,089 hospital-treated patients and 424 patients remaining in the community. Updated projections were produced for two scenarios starting on 1 December. In the first scenario, we assumed future hospitalization rate to be drawn from the revised set of plausible historical rates. In the second scenario, we assumed the future hospitalization rate to be fixed at 85% (corresponding to Scenario C in the S1 Text). In both scenarios, hospital capacity was represented as the sum of the projected capacity of ETUs, community care units, and holding units in operation in the country at the given time, obtained from reports by the UN Mission for Ebola Emergency Response and International Red Cross projections.

**Table 3:** Mean values and inter-quartile ranges of plausible parameter sets.

**Variable**	**Mean (Original)**	**IQR (Original)**	**Mean (Updated)**	**IQR (Updated)**
Hospitalization rate (*h*)	0.6	(0.53–0.68)	0.72	(0.71–0.73)
Overdispersion (*θ*)	2.2	(2.0–2.3)	2.2	(2.0–2.4)
Secondary cases from a funeral (*ϕ*)	5.9	(5.2–6.6)	6.1	(5.6–6.7)
Maximum secure burial rate (γ_1_)	0.6	(0.53–0.66)	0.68	(0.65–0.71)
Secure burial improvement (γ_2_)	2.0	(1.8–2.2)	1.9	(1.7–2.1)
Core secondary transmission rate (*λ*)	1.1	(1.0–1.2)	1.1	(1.0–1.2)
Hospital leakage (*λ_h_*)	0.25	(0.22–0.28)	0.25	(0.21–0.27)

S12 Fig. shows results are robust to the range of the latin hypercube sample.

IQR, inter-quartile range.

## Results

### Model Fit

Overall, 1,045 of 5,000 (20.9%) parameter sets were initially determined to be plausible under the data available to 2 September. This total was reduced to 46 parameter sets in the updated model using data to 1 December. Mean values and inter-quartile range from plausible parameter sets are reported in Table 3. The parameter that showed the greatest change between the initial parameterization and updated model was the hospitalization rate, which the updated model estimated to be greater than 70% whereas earlier in the year it was estimated to be around 60%. Parameter correlations are shown in S13–S15 Figs.

**Figure 3:**
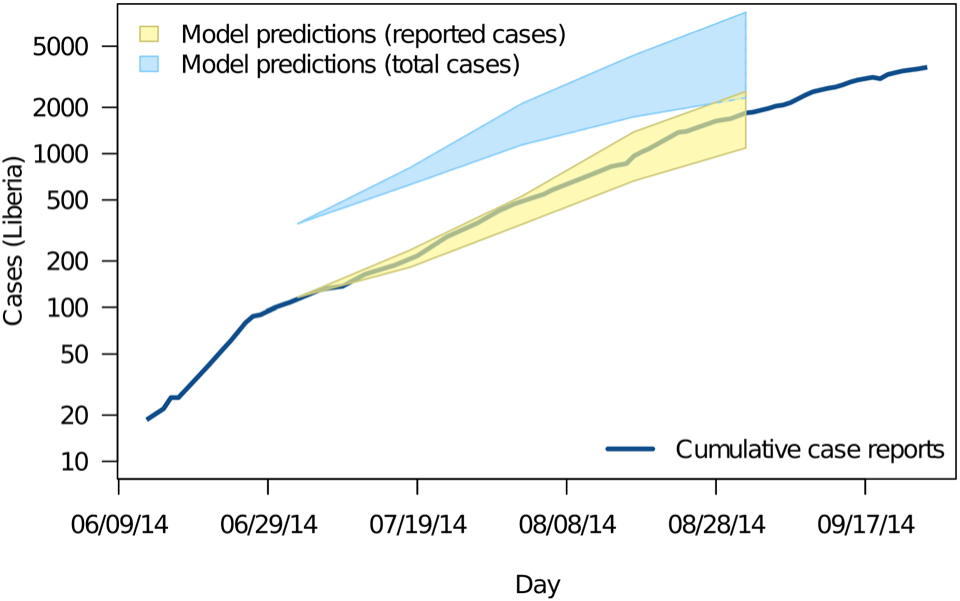
The fit model for Ebola transmission in Liberia initialized on 4 July. The heavy blue line shows the cumulative number of cases reported. The yellow region shows the model-predicted range of cases expected to be reported given incomplete reporting. The blue region shows the model-predicted total number of cases over the same time. The underlying data and code to generate this figure may be obtained by running the file “ebola-forecasting-main-text.R” deposited in the Dryad repository: http://doi.org/10.5061/dryad.17m5q.

**Figure 4:**
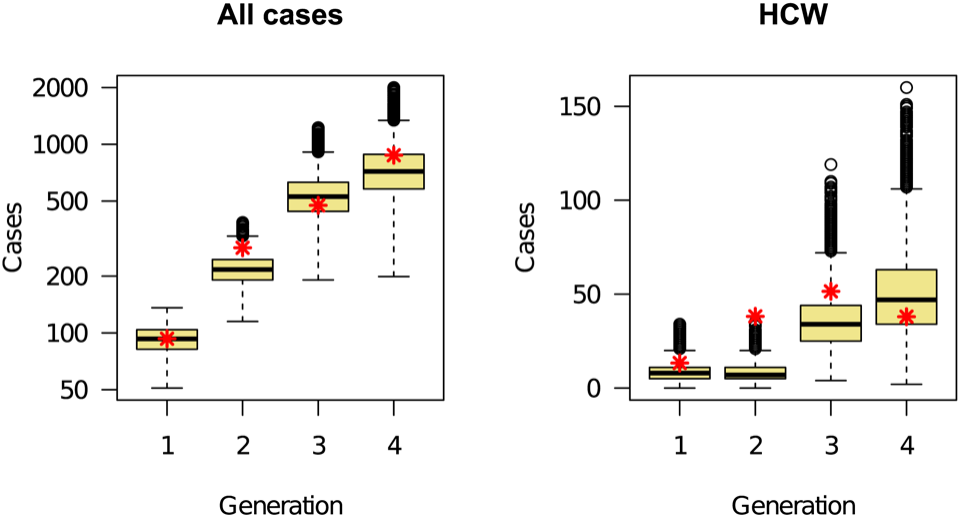
Comparison between the total number of reported cases (aggregated data from Table 2, red asterisks) and model-generated distribution (box-and-whisker plots) during four generations of the epidemic, starting 4 July 2014. Shown are values for all cases (left panel) and cases among HCWs (right panel). The underlying data and code to generate this figure may be obtained by running the file “ebola-forecasting-main-text.R” deposited in the Dryad repository: http://doi.org/10.5061/dryad.17m5q.

The fit between the ensemble of plausible parameterizations and the cumulative number of reported cases in Liberia during the interval used for model fitting is shown in Fig. 3. The heavy blue line shows the cumulative number of reported cases. The plausible range of case reports given the model is shown in yellow (95% prediction intervals). The plausible range of total cases, including unreported cases, is shown in blue. The fit of the model to infection generations in HCWs and in the community is shown in Fig. 4. These figures show that the initial model reliably reproduced the observed epidemic trajectory at the time the model was produced.

### Effective Reproduction Number

**Figure 5:**
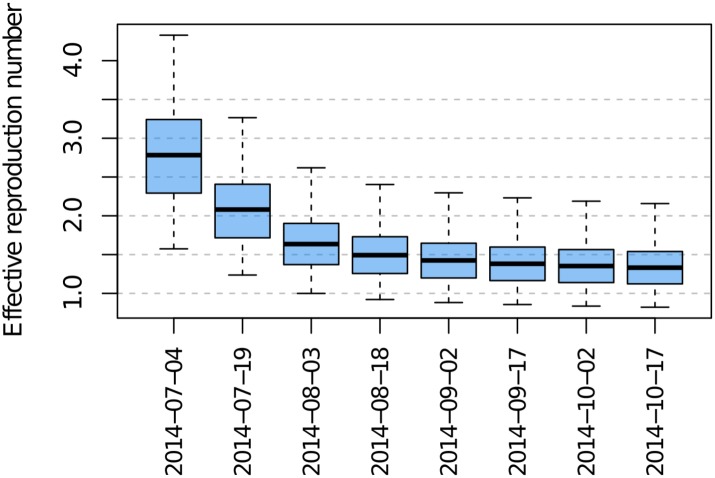
Effective reproduction numbers for Ebola virus in Liberia from July–October 2014. The underlying data and code to generate this figure may be obtained by running the file “ebola-forecasting-main-text.R” deposited in the Dryad repository: http://doi.org/10.5061/dryad.17m5q.

Model-based effective reproduction numbers at infection generations between 4 July and 17 October were calculated by evaluating the effective reproduction number (see S1 Text) at the 1,045 plausible parameter sets. The change over time in the range of plausible effective reproduction numbers is shown in Fig. 5.

### Forecasts and Containment

**Figure 6:**
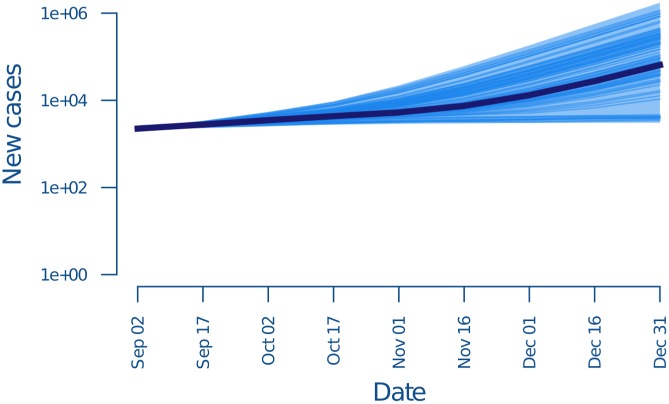
Number of cases in each infection generation when transmission occurs at the baseline rate as predicted by the data available by September 2014. Dark blue lines show 100 realizations of the stochastic model. Shaded region shows 95% quantiles over 10,450 realizations; *x*-axis is the final date of the infection generation, e.g., 17 September is for all cases in the infection generation beginning 2 September. The underlying data and code to generate this figure may be obtained by running the file “ebola-forecasting-main-text.R” deposited in the Dryad repository: http://doi.org/10.5061/dryad.17m5q.

Simulated trajectories illustrating the possible outcomes starting on 2 September, using data only up to that point and assuming baseline conditions, are shown in Fig. 6. The median projected total epidemic size by 31 December was 130,862 cases (inter-quartile range: 44,560–396,706). The top panel shows the range of trajectories for 10,450 simulations distributed over 1,045 plausible parameter sets. An interpolation to project the daily number of persons seeking hospitalization is shown in S2 Fig. Simulated trajectories for other scenarios are shown in S3, S5, S7, and S9 Figs. Daily number of persons seeking hospitalization for these scenarios are shown in S4, S6, S8, and S10 Figs. Scenarios are compared in S12 Fig. These results show that given the best information available in October 2014, it was reasonable to conclude that the total number of cases might exceed 100,000 by the end of the 2014 calendar year.

**Figure 7:**
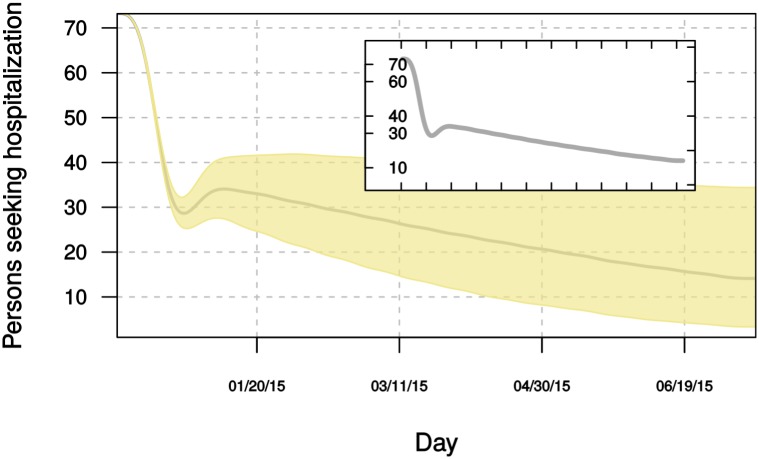
Projected daily hospital demand in 2015 for Ebola patients in Liberia if hospitalization rates are the same as in July–September Summer 2014. Gray line and inset plot show the median daily number of cases. The underlying data and code to generate this figure may be obtained by running the file “ebola-forecasting-main-text.R” deposited in the Dryad repository: http://doi.org/10.5061/dryad.17m5q.

**Figure 8:**
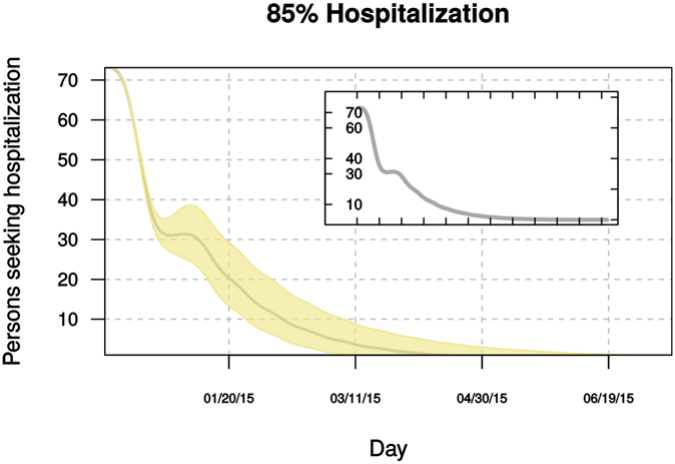
Projected daily hospital demand in 2015 for Ebola patients in Liberia if 85% of cases are hospitalized. Gray line and inset plot show the median daily number of cases. The underlying data and code to generate this figure may be obtained by running the file “ebola-forecasting-main-text.R” deposited in the Dryad repository: http://doi.org/10.5061/dryad.17m5q.

Projections from 1 December 2014 to 13 July 2015, fit using data up to 1 December, are shown in Figs. 7 and 8. These simulations, accounting for information available to 1 December, show that interventions and changes to personal behavior substantially reduced transmission compared with earlier in the year. However, these results suggest that transmission could remain “near critical” (*R_eff_* ≈1) if rates of patient hospitalization estimated to have occurred in July–September 2014 are maintained (Fig. 7). In this scenario, active transmission would almost certainly continue into the second half of 2015. By contrast, if patient hospitalization of 85% can be achieved, simulations suggest the epidemic will be largely contained sometime between March and June 2015 (Fig. 8). Both scenarios predict a rapid decline in the first months of 2015, followed by a longer “tail.”

## Discussion

The transmission of Ebola virus in West Africa continues to give rise to high mortality and morbidity. Part of the challenge in predicting the progression of the epidemic lies in the fundamentally different ways in which transmission occurs: infection of hospital workers, community care-givers, and those preparing bodies for funerals [1]. Additionally, the time frame and effectiveness of increased hospital capacity compounds the problem of prediction [35], whether it is aimed at anticipating demand for hospitalization or determining the level and speed of intervention needed to bring the outbreak under control. Our approach was to represent heterogeneity in transmission and time-varying intervention in a multi-type branching process model [36] that offers analytic tractability, efficient simulation, and the flexibility to investigate a wide range of intervention scenarios. It is closely related to sources of data; for example, stratifying cases into hospital-treated versus community-treated allows for estimating under-reporting, which is thought to be large for the current epidemic [37].

Analytical insight, particularly the derivation of a reproductive ratio, is useful when parameter estimates (such as the hospitalization rate) are uncertain, since the sign and magnitude of their effects on transmission can be derived. Besides recovering a full expression for the basic reproductive ratio, simplifying assumptions such as assuming that funeral-associated transmission can be reduced to zero, yield further understanding. In particular, our model shows how the additional exposure to HCWs in a hospital environment (*β*) combines with both the reduced transmission in that environment (*α*) and the hospitalization rate (*h*) to determine when community (versus hospital) transmission will dominate (i.e., when 1−*h>αβ*). Such formulas may provide “rules of thumb” to help guide infection control or could improve practical decision making by regularly updating estimates of core parameters through surveillance within health facilities.

The approach we have taken to model parameterization is novel. A more familiar approach is to propose a deterministic or stochastic model that is then fit by minimizing an objective function on the errors, e.g., sum of squared errors or negative log likelihood of the data given the model [29]. Statistical interpretation of such models (such as hypothesis tests or confidence intervals) relies heavily on the parametric specification of both the process model and the observation model. If the proposed models are not good approximations to their respective contributions to the data-generating process (that is, they have considerable “structural error”), then these quantities may be quite biased. Moreover, such models are ineffective when they are overparameterized. Our approach—the construction of plausible parameter sets that are both epidemiologically sensible and can reproduce observed properties of the epidemic—seeks instead to understand the space of models consistent with the data. The cost of this approach is that the results do not admit probabilistic interpretations, hypothesis tests, or traditional confidence intervals. A byproduct is that the identifiability of parameters (which is compromised by overparameterization) is no longer an obstacle to model construction and forecasting. If two parameters, say *a* and *b*, are highly correlated (not simultaneously identifiable) so that either the model with large *a* and small *b* or large *b* and small *a* are both consistent with the data, then the plausible set will include parameter combinations with examples of both kinds (but not, for example, large *a* and large *b* or small *a* and small *b*). It may be that these differences are in fact irrelevant to the eventual behavior of the model, in which case the space of possible solutions will be small. Alternatively, it may be that these are just the parameters that most substantially influence alternative outcomes, in which case the space of possible solutions will be large. By seeking bounds on the range of outcomes rather than a unique causal story, the method of plausible parameter sets avoids technical problems with model identifiability and more accurately emphasizes the kind of uncertainty prevalent under emergent conditions while focusing attention on the property of most practical interest: the possible future trajectories of the epidemic. In conclusion, we believe that the method of plausible parameter sets is a good starting point for exploring entire families of models and for setting bounds on the range of possible outcomes. It is a first step toward the construction of models for probabilistic inference.

In this study, we have focused on Liberia, which initially experienced the fastest epidemic growth. The ramping up of hospital capacity in Liberia was dramatic during late August 2014, adding approximately 300 beds. Throughout September, that sustained effort led to an additional ~300 beds. This heterogeneous increase in capacity over time was incorporated into our model. We investigated alternative hospital capacities and demands in a set of plausible alternative scenarios. The best and worse outcomes of these scenarios vary dramatically in the forecasted epidemic size (S12 Fig). Median estimates were at around 130,000 cases by 31 December 2014 assuming a baseline scenario without increased hospital capacity. This was reduced to around 50,000 when capacity was ramped up to ~1,700. Further increases in hospital capacity were shown to reduce the upper bound on our predictions, but did not substantially affect the median. Our initial model suggested that if the hospitalization rate could be increased to 85% then it was probable that the epidemic would be contained. The updated model confirms this result and predicts near elimination sometime between March and June of 2015. The updated model also highlights the continued need for vigilance, however, suggesting that if hospitalization returns to prior levels the current outbreak may exhibit an extremely prolonged right tail. In conclusion, these modeling exercises suggested that in the absence of rapid hospitalization of most cases, none of the proposed scenarios for increasing hospital capacity would have been likely to achieve containment. Continuing on the path to elimination will require sustained watchfulness and individual willingness to be treated.

Although broadly consistent with our narrative understanding of the epidemiology of Ebola virus disease in West Africa, the model we developed does not account for some known features of transmission, mainly because we believe these effects to be small relative to the processes represented. For instance, the size of the at-risk population of HCWs has varied over time, which may account for some of the fluctuations in infection within this group of people (Fig. 4). Our model assumes that the number of contacts between HCWs and infected persons is proportional to the number of infected persons limited by hospital capacity. To the extent that individual care was reduced because of exhaustion or movement of the care-giving workforce early in the epidemic, our model is unrealistic. Individual case information will be required to determine the magnitude of this effect. Similarly, our model attributes the decline in transmission primarily to hospitalization and safe burial, but not improved infection control in the hospital setting, better and safer use of personal protective equipment, or social distancing. We believe that infection control and effective use of protective equipment are in fact key elements to containing Ebola and may account for some of the proportional decline in transmission to HCWs shown in Fig. 4. Changes in transmission in the hospital environment were not included in our model for the technical reason that time-varying hospital transmission and time-varying safe burial could not be simultaneously estimated together with evidence that (i) by late summer, transmission to HCWs was a small fraction of transmission overall, and (ii) our model already attributes a high level of effectiveness to infection control (see section “Transmission and the effectiveness of infection control”). Effects of social distancing are probably captured numerically by our model in the estimated decline in funerary transmission. To the extent that transmission has been reduced by diffuse social distancing—including the use of safety precautions in households of infected persons—our estimate of the safe burial rate will be biased. The upshot is that our model may be numerically accurate, although *g* may not reflect the true safe burial rate. To the extent that declines in transmission are due to changes other than increased hospital capacity and safe burial, the projected benefits of future increases in hospital capacity may be exaggerated. In this respect, our forecasts are conjectures based on current understanding.

Branching process models use offspring distributions to simulate forward in time. Here, the offspring of an infectious individual refers to the new cases generated from that infectious individual. This is the type of data that is frequently reported, even during early stages of an outbreak. Models that require separate quantities for the probability of infection and number of contacts are complicated by the fact that there is uncertainty about whether contact is effective or not. For example, how many “contacts” of an infectious individual transported by airplane are sufficiently intimate that infection is even a causal possibility? Ambiguities about the causal relevance of contacts of different kinds complicate models expressed in terms of attack rates. By focusing on the empirical offspring distributions in various transmission settings, one is able to build, simulate, and analyze a model with the key epidemiological features, and to investigate a wide range of mitigation scenarios. In our case, the result was a multi-type branching process that separated the location that infection was acquired from the sites generating new infections. This approach captures the behavioral aspects of transmission that are often lacking in models [38]. Awareness of Ebola in the community and public education mean that community-acquired transmission is increasingly likely to lead to demand for hospitalization. While our methods are focused on the current Ebola outbreak in West Africa, they apply to a broad class of infectious diseases.

## Supporting Information

S1 Fig Cumulative reported cases in data (red lines) and model simulations (box-and-whisker plots). The left three panels show results for HCWs, reported cases, and reported and unreported cases (assuming 2.5-fold under-reporting). The remaining panels show the model-predicted distributions of hospital-acquired infections and funeral-acquired infections. The underlying data and code to generate this figure may be obtained by running the file “ebola-forecasting-supplement.R” deposited in the Dryad repository: http://doi.org/10.5061/dryad.17m5q.
(PDF)

S2 Fig Daily number of persons seeking hospitalization (bottom) when transmission occurs at the baseline rate. Inset plot shows the median daily number of cases. The underlying data and code to generate this figure may be obtained by running the file “ebola-forecasting-supplement.R” deposited in the Dryad repository: http://doi.org/10.5061/dryad.17m5q.
(PDF)

S3 Fig Number of cases in each infection generation under Scenario A. The underlying data and code to generate this figure may be obtained by running the file “ebola-forecasting-supplement.R” deposited in the Dryad repository: http://doi.org/10.5061/dryad.17m5q.
(PDF)

S4 Fig Daily number of persons seeking hospitalization (bottom) according to Scenario A. Inset plot shows the median daily number of cases. The underlying data and code to generate this figure may be obtained by running the file “ebola-forecasting-supplement.R” deposited in the Dryad repository: http://doi.org/10.5061/dryad.17m5q.
(PDF)

S5 Fig Number of cases in each infection generation under Scenario B. The underlying data and code to generate this figure may be obtained by running the file “ebola-forecasting-supplement.R” deposited in the Dryad repository: http://doi.org/10.5061/dryad.17m5q.
(PDF)

S6 Fig Daily number of persons seeking hospitalization (bottom) according to Scenario B. Inset plot shows the median daily number of cases. The underlying data and code to generate this figure may be obtained by running the file “ebola-forecasting-supplement.R” deposited in the Dryad repository: http://doi.org/10.5061/dryad.17m5q.
(PDF)

S7 Fig Number of cases in each infection generation under Scenario C. The underlying data and code to generate this figure may be obtained by running the file “ebola-forecasting-supplement.R” deposited in the Dryad repository: http://doi.org/10.5061/dryad.17m5q.
(PDF)

S8 Fig Daily number of persons seeking hospitalization (bottom) according to under Scenario C. Inset plot shows the median daily number of cases. The underlying data and code to generate this figure may be obtained by running the file “ebola-forecasting-supplement.R” deposited in the Dryad repository: http://doi.org/10.5061/dryad.17m5q.
(PDF)

S9 Fig Number of cases in each infection generation under Scenario D. The underlying data and code to generate this figure may be obtained by running the file “ebola-forecasting-supplement.R” deposited in the Dryad repository: http://doi.org/10.5061/dryad.17m5q.
(PDF)

S10 Fig Daily number of persons seeking hospitalization (bottom) according to under Scenario D. Inset plot shows the median daily number of cases. The underlying data and code to generate this figure may be obtained by running the file “ebola-forecasting-supplement.R” deposited in the Dryad repository: http://doi.org/10.5061/dryad.17m5q.
(PDF)

S11 Fig Cumulative distribution function (left) and histogram (right) of the total epidemic size (top) and epidemic duration in days after 2 September 2014 (bottom) in a containment scenario (Scenario D). The underlying data and code to generate this figure may be obtained by running the file “ebola-forecasting-supplement.R” deposited in the Dryad repository: http://doi.org/10.5061/dryad.17m5q.
(PDF)

S12 Fig Robustness of the method of plausible parameter sets. Panels show the distribution of number of cases by December 31, 2014 in five scenarios. Scenario A reflects increased hospital capacity from US Department of Defense (DoD) commitment of 15 September. Scenario B assumed significantly increased hospital capacity in excess of Scenario A. Scenario C reflects significantly increased hospital capacity and increased hospitalization rates. Scenario D reflects significantly increased hospital capacity and significantly increased hospitalization. Light blue shaded regions show outcomes from the latin hypercube neighborhood of ±25% of the best fit values. Gray lines show the range of outcomes from a larger parameter space (latin hypercube sampling within ±50% of the least squares estimates). Dark blue lines show the range of outcomes from a smaller parameter space (latin hypercube sampling within ±10% of the least squares estimates). These simulations show that even very different endpoints to the sampled parameter region do not change the primary qualitative conclusions of this study. The underlying data and code to generate this figure may be obtained by running the file “ebola-forecasting-supplement.R” deposited in the Dryad repository: http://doi.org/10.5061/dryad.17m5q.
(PDF)

S13 Fig Parameter pairs in plausible parameter sets obtained from a latin hypercube sample in a neighborhood ±25% of the estimated values. Correlation coefficients (absolute value) are shown in the subdiagonal plots; values greater than 0.25 are highlighted in blue. The underlying data and code to generate this figure may be obtained by running the file “ebola-forecasting-supplement.R” deposited in the Dryad repository: http://doi.org/10.5061/dryad.17m5q.
(PDF)

S14 Fig Parameter pairs in plausible parameter sets obtained from a latin hypercube sample in a neighborhood ±10% of the estimated values. Correlation coefficients (absolute value) are shown in the subdiagonal plots; values greater than 0.25 are highlighted in blue. The underlying data and code to generate this figure may be obtained by running the file “ebola-forecasting-supplement.R” deposited in the Dryad repository: http://doi.org/10.5061/dryad.17m5q.
(PDF)

S15 Fig Parameter pairs in plausible parameter sets obtained from a latin hypercube sample in a neighborhood ±50% of the estimated values. Correlation coefficients (absolute value) are shown in the subdiagonal plots; values greater than 0.25 are highlighted in blue. The underlying data and code to generate this figure may be obtained by running the file “ebola-forecasting-supplement.R” deposited in the Dryad repository: http://doi.org/10.5061/dryad.17m5q.
(PDF)

S1 Text Expression for the effective reproduction number and supplementary analysis. (PDF)

## Abbreviations

ETU: Ebola treatment unit
HCW: health care worker
MSF: Médecins Sans Frontières
