# Supplementary figures and images for: Ebola Cases and Health System Demand in Liberia
Source: PLoS Biol. 2015 Jan 13;13(1):e1002056. doi: 10.1371/journal.pbio.1002056 (PMC4293091; doi:10.1371/journal.pbio.1002056)

log HCW

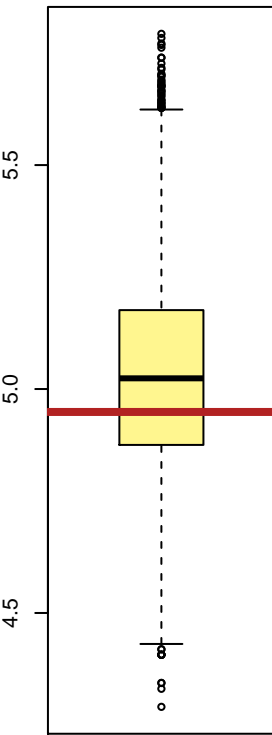

log Total reports

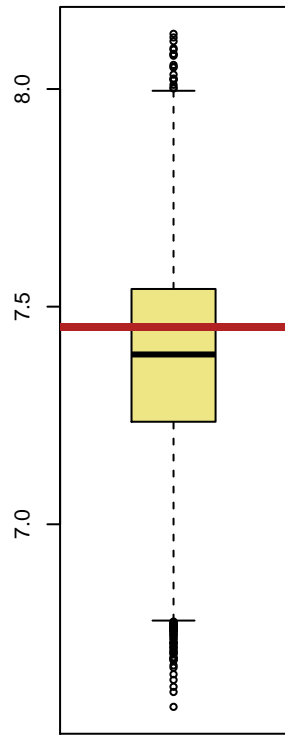

log Total cases

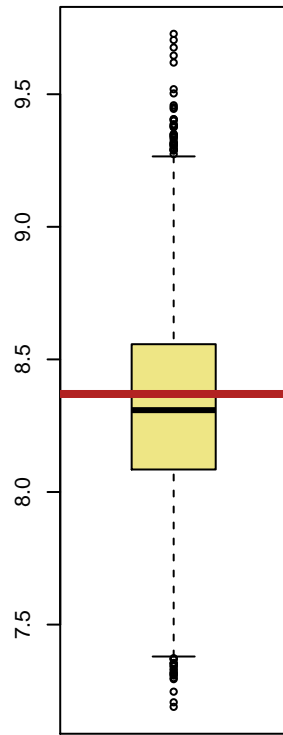

Hospital acquired

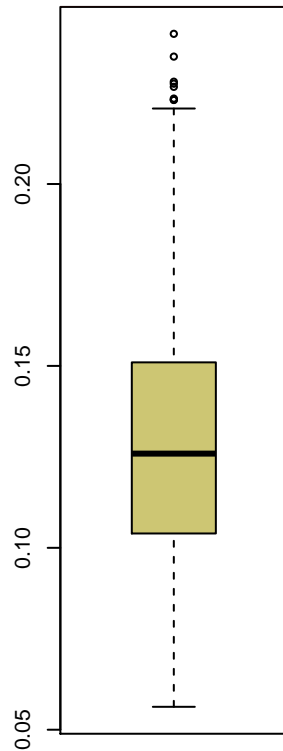

Funeral acquired

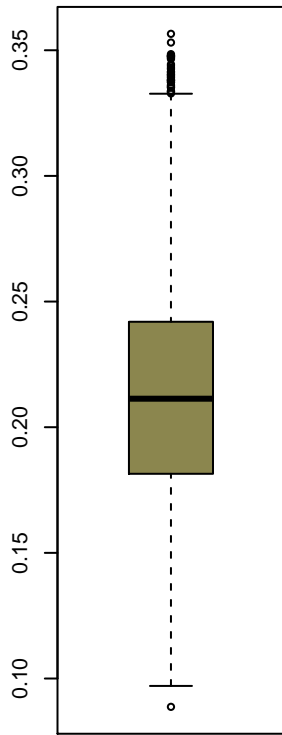

Supplement: S1 Fig — The left three panels show results for HCWs, reported cases, and reported and unreported cases (assuming 2.5-fold under-reporting). The remaining panels show the model-predicted distributions of hospital-acquired infections and funeral-acquired infections. The underlying data and code to generate this figure may be obtained by running the file “ebola-forecasting-supplement.R” deposited in the Dryad repository: http://doi.org/10.5061/dryad.17m5q. (PDF) [file pbio.1002056.s001.pdf]

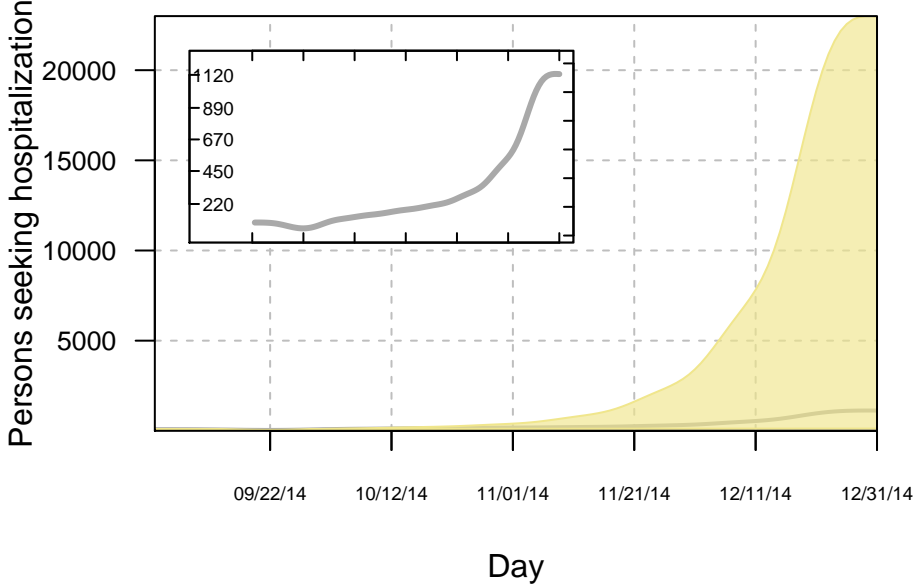

Supplement: S2 Fig — Inset plot shows the median daily number of cases. The underlying data and code to generate this figure may be obtained by running the file “ebola-forecasting-supplement.R” deposited in the Dryad repository: http://doi.org/10.5061/dryad.17m5q. (PDF) [file pbio.1002056.s002.pdf]

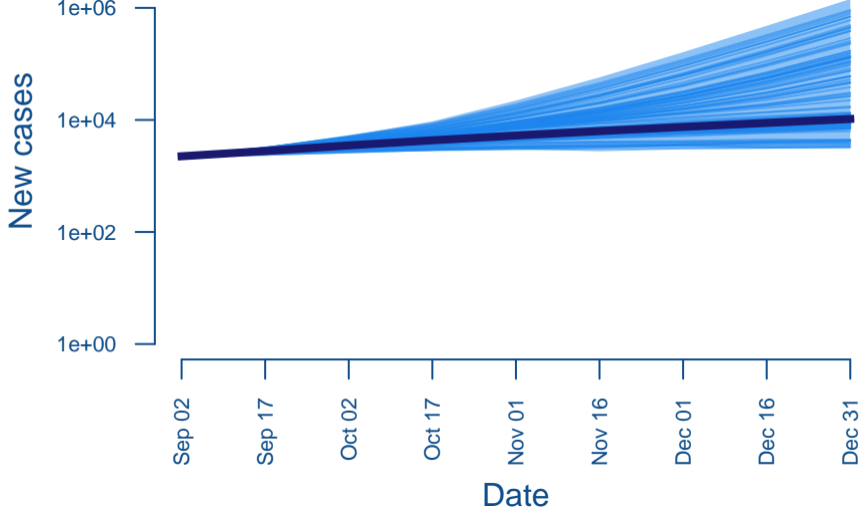

Supplement: S3 Fig — The underlying data and code to generate this figure may be obtained by running the file “ebola-forecasting-supplement.R” deposited in the Dryad repository: http://doi.org/10.5061/dryad.17m5q. (PDF) [file pbio.1002056.s003.pdf]

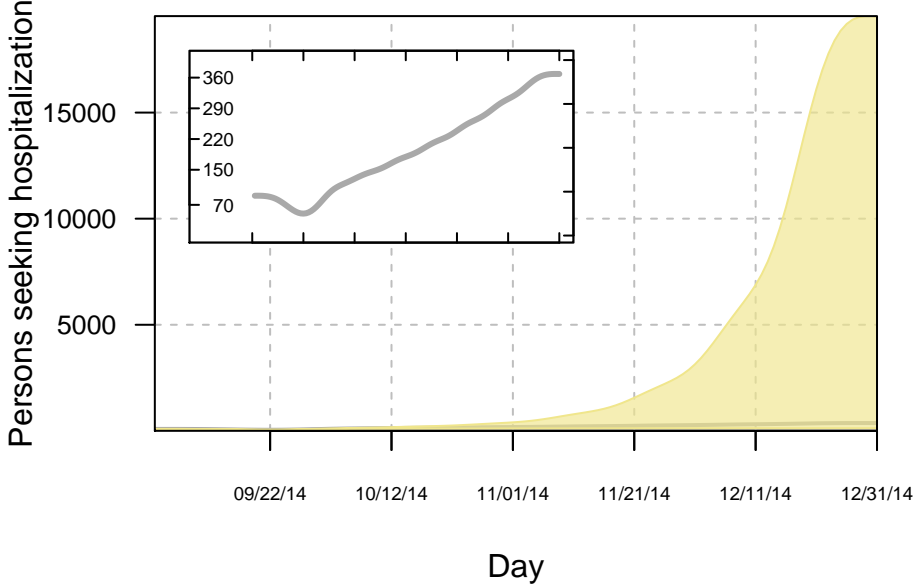

Supplement: S4 Fig — Inset plot shows the median daily number of cases. The underlying data and code to generate this figure may be obtained by running the file “ebola-forecasting-supplement.R” deposited in the Dryad repository: http://doi.org/10.5061/dryad.17m5q. (PDF) [file pbio.1002056.s004.pdf]

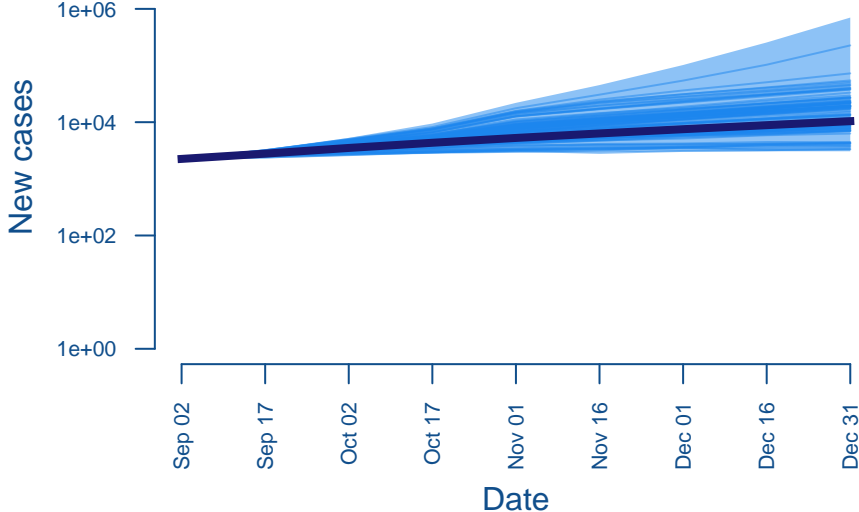

Supplement: S5 Fig — The underlying data and code to generate this figure may be obtained by running the file “ebola-forecasting-supplement.R” deposited in the Dryad repository: http://doi.org/10.5061/dryad.17m5q. (PDF) [file pbio.1002056.s005.pdf]

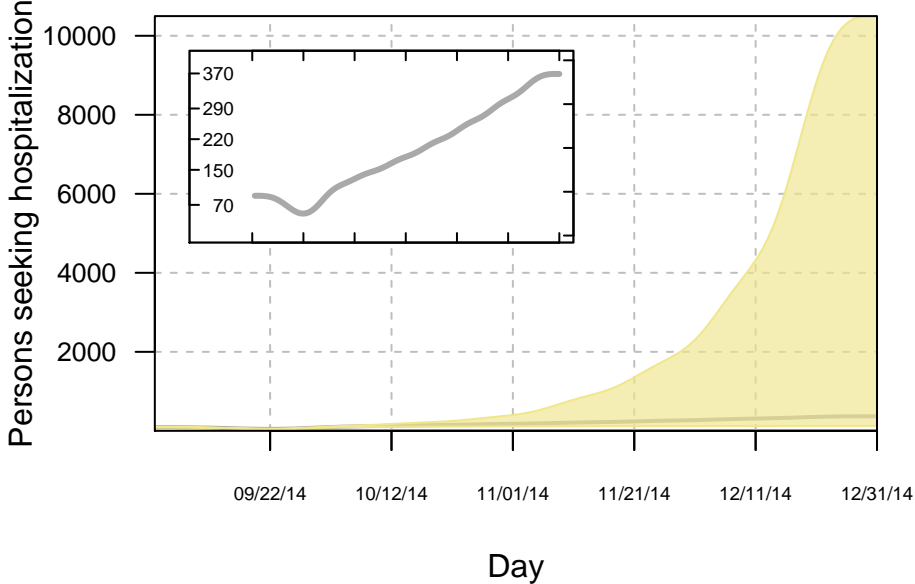

Supplement: S6 Fig — Inset plot shows the median daily number of cases. The underlying data and code to generate this figure may be obtained by running the file “ebola-forecasting-supplement.R” deposited in the Dryad repository: http://doi.org/10.5061/dryad.17m5q. (PDF) [file pbio.1002056.s006.pdf]

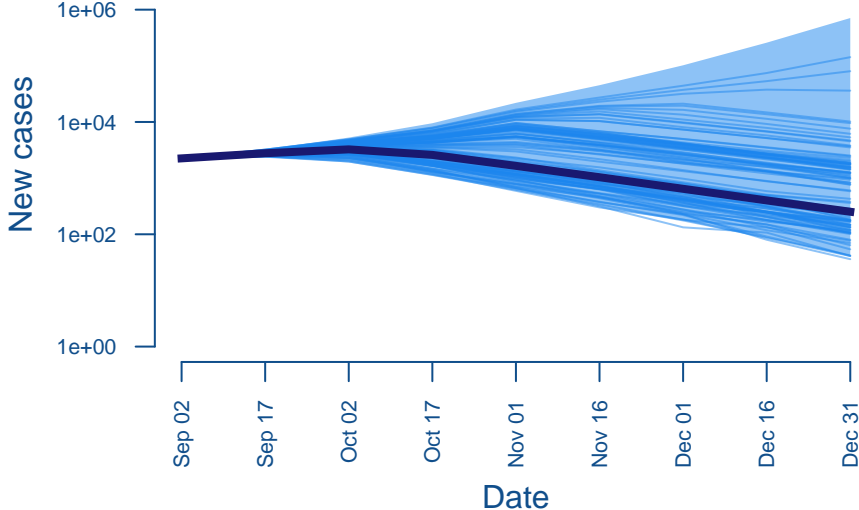

Supplement: S7 Fig — The underlying data and code to generate this figure may be obtained by running the file “ebola-forecasting-supplement.R” deposited in the Dryad repository: http://doi.org/10.5061/dryad.17m5q. (PDF) [file pbio.1002056.s007.pdf]

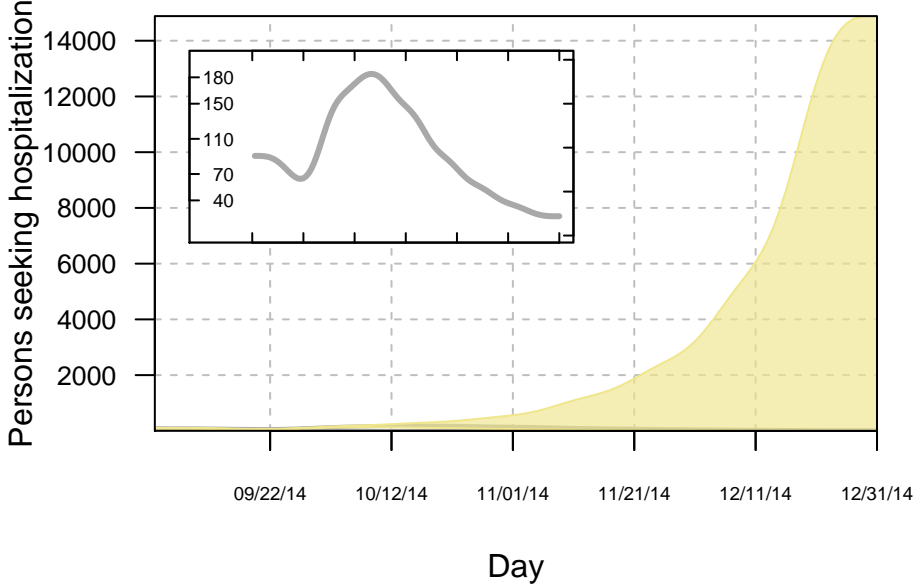

Supplement: S8 Fig — Inset plot shows the median daily number of cases. The underlying data and code to generate this figure may be obtained by running the file “ebola-forecasting-supplement.R” deposited in the Dryad repository: http://doi.org/10.5061/dryad.17m5q. (PDF) [file pbio.1002056.s008.pdf]

New cases

1e+08  
1e+06  
1e+04  
1e+02  
1e+00

Sep 02

Sep 17

Oct 02

Oct 17

Nov 01

Nov 16

Dec 01

Dec 16

Dec 31

Jan 15

Jan 30

Feb 14

Mar 01

Mar 16

Date

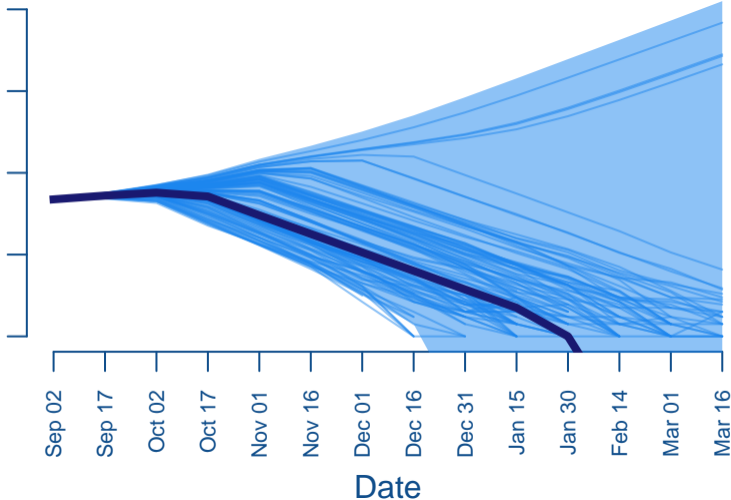

Supplement: S9 Fig — The underlying data and code to generate this figure may be obtained by running the file “ebola-forecasting-supplement.R” deposited in the Dryad repository: http://doi.org/10.5061/dryad.17m5q. (PDF) [file pbio.1002056.s009.pdf]

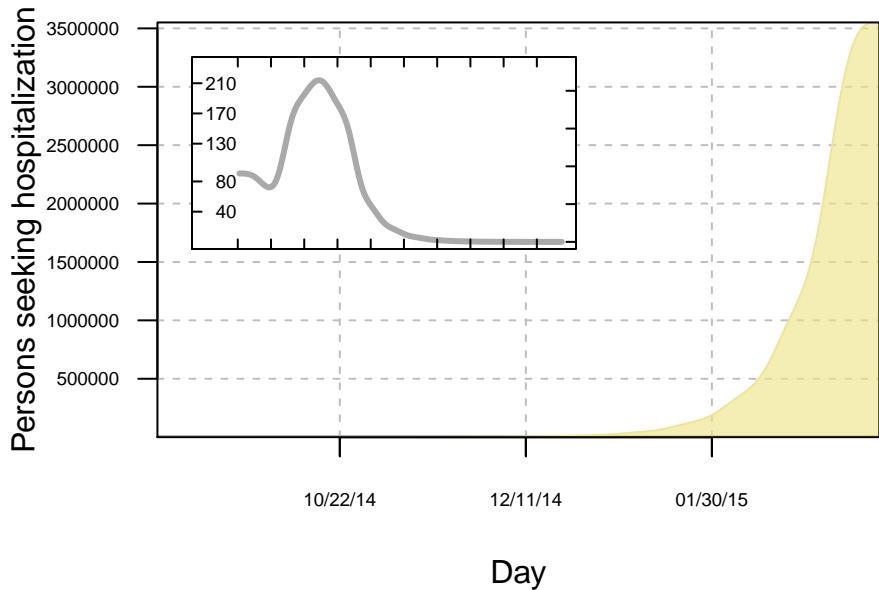

Supplement: S10 Fig — Inset plot shows the median daily number of cases. The underlying data and code to generate this figure may be obtained by running the file “ebola-forecasting-supplement.R” deposited in the Dryad repository: http://doi.org/10.5061/dryad.17m5q. (PDF) [file pbio.1002056.s010.pdf]

# Projected epidemic size by end of 2014

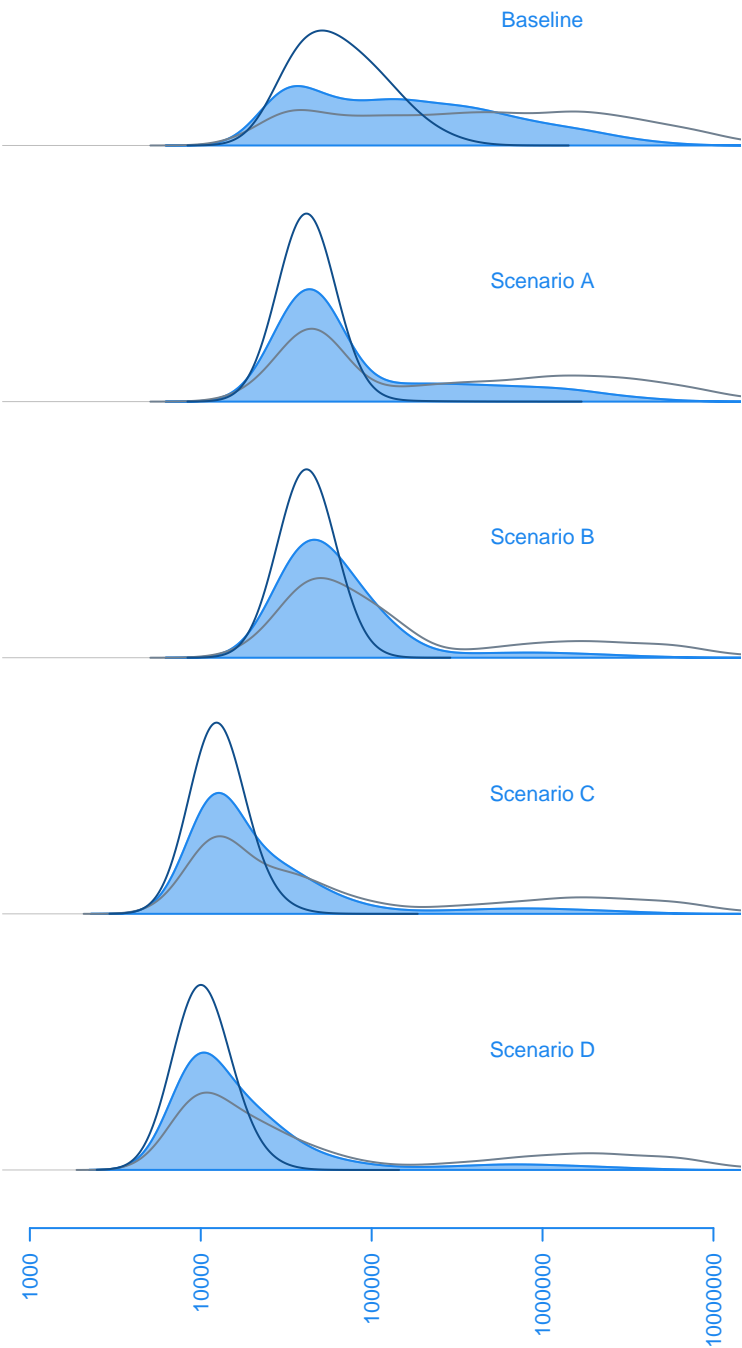

Supplement: S12 Fig — Panels show the distribution of number of cases by December 31, 2014 in five scenarios. Scenario A reflects increased hospital capacity from US Department of Defense (DoD) commitment of 15 September. Scenario B assumed significantly increased hospital capacity in excess of Scenario A. Scenario C reflects significantly increased hospital capacity and increased hospitalization rates. Scenario D reflects significantly increased hospital capacity and significantly increased hospitalization. Light blue shaded regions show outcomes from the latin hypercube neighborhood of ±25% of the best fit values. Gray lines show the range of outcomes from a larger parameter space (latin hypercube sampling within ±50% of the least squares estimates). Dark blue lines show the range of outcomes from a smaller parameter space (latin hypercube sampling within ±10% of the least squares estimates). These simulations show that even very different endpoints to the sampled parameter region do not change the primary qualitative conclusions of this study. The underlying data and code to generate this figure may be obtained by running the file “ebola-forecasting-supplement.R” deposited in the Dryad repository: http://doi.org/10.5061/dryad.17m5q. (PDF) [file pbio.1002056.s012.pdf]

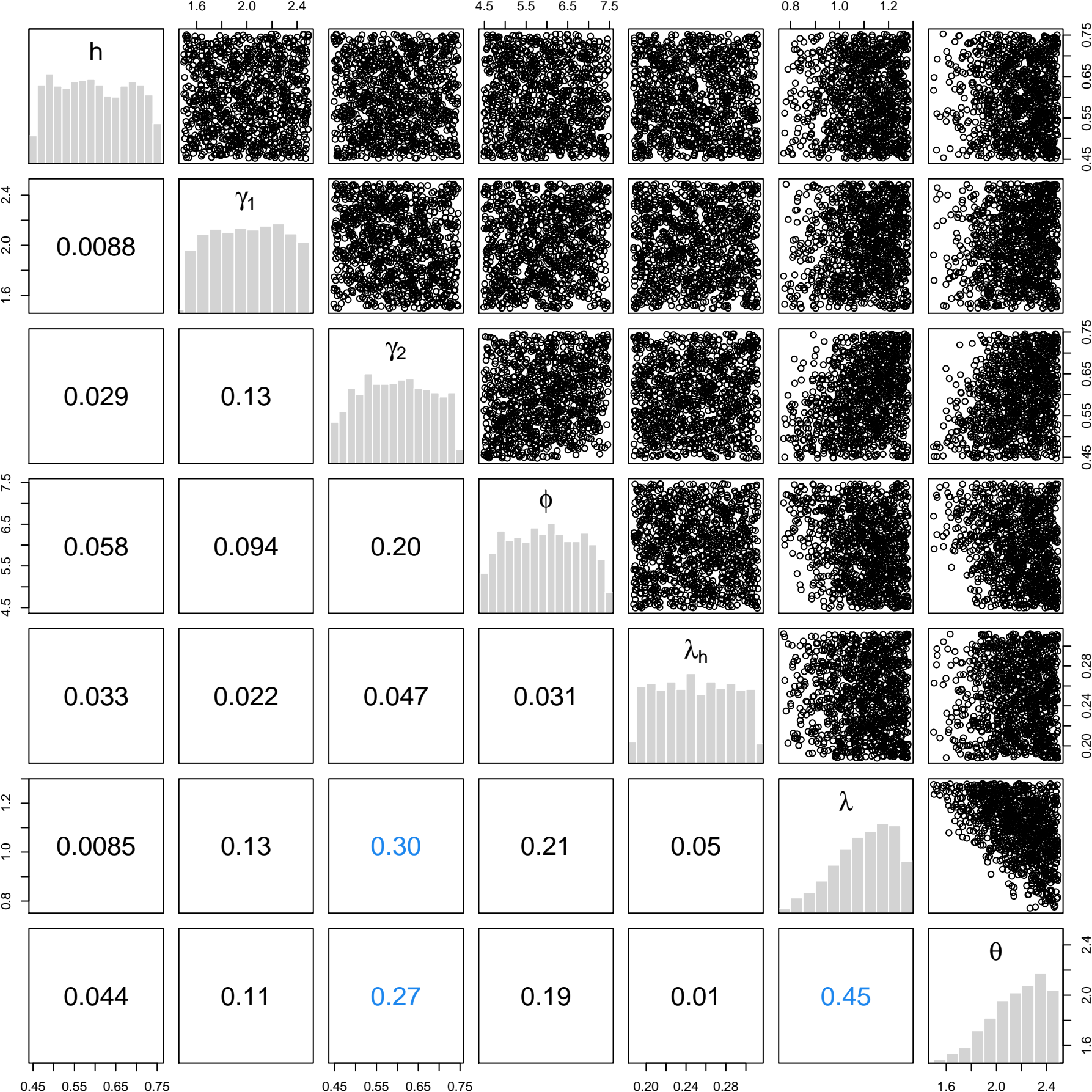

Supplement: S13 Fig — Correlation coefficients (absolute value) are shown in the subdiagonal plots; values greater than 0.25 are highlighted in blue. The underlying data and code to generate this figure may be obtained by running the file “ebola-forecasting-supplement.R” deposited in the Dryad repository: http://doi.org/10.5061/dryad.17m5q. (PDF) [file pbio.1002056.s013.pdf]

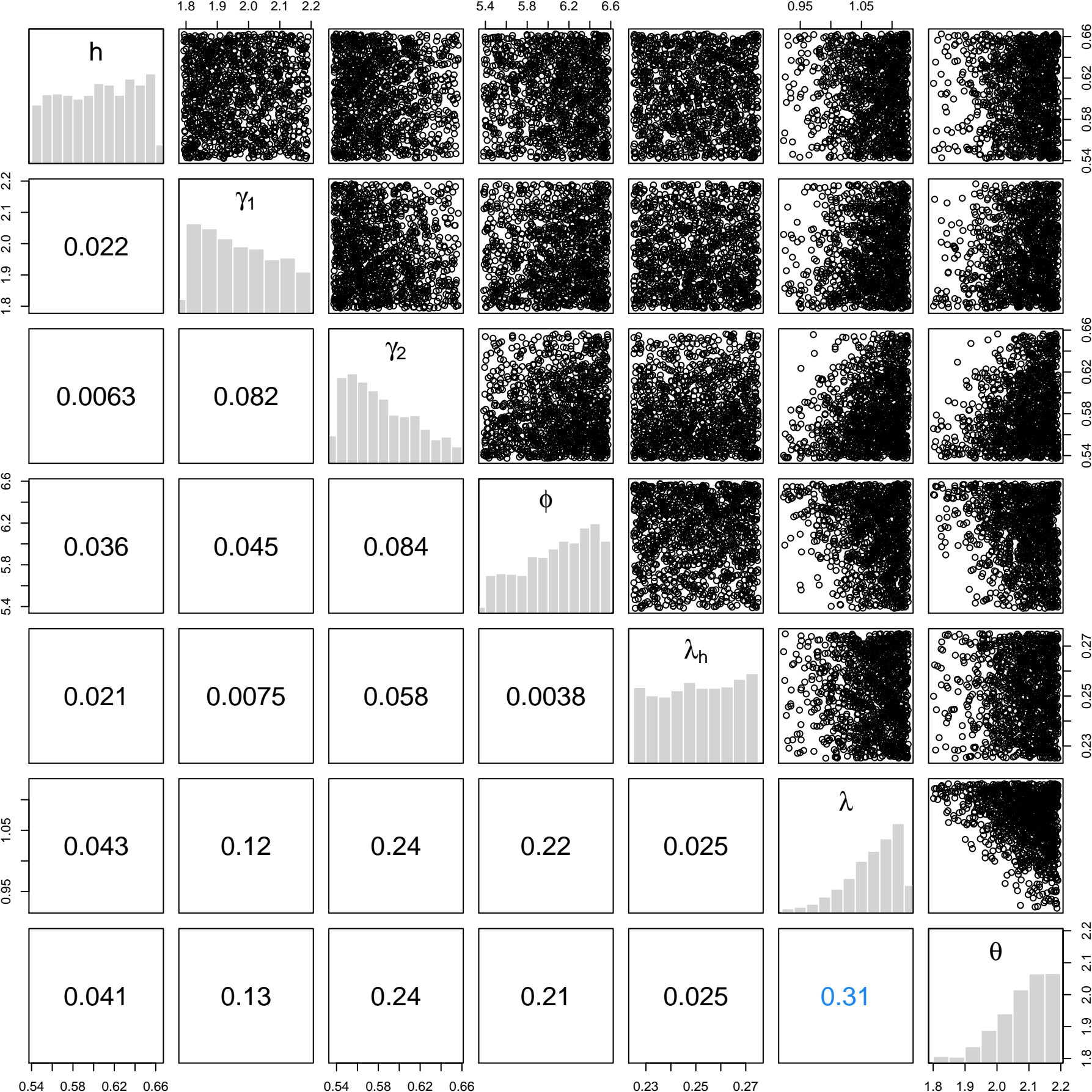

Supplement: S14 Fig — Correlation coefficients (absolute value) are shown in the subdiagonal plots; values greater than 0.25 are highlighted in blue. The underlying data and code to generate this figure may be obtained by running the file “ebola-forecasting-supplement.R” deposited in the Dryad repository: http://doi.org/10.5061/dryad.17m5q. (PDF) [file pbio.1002056.s014.pdf]

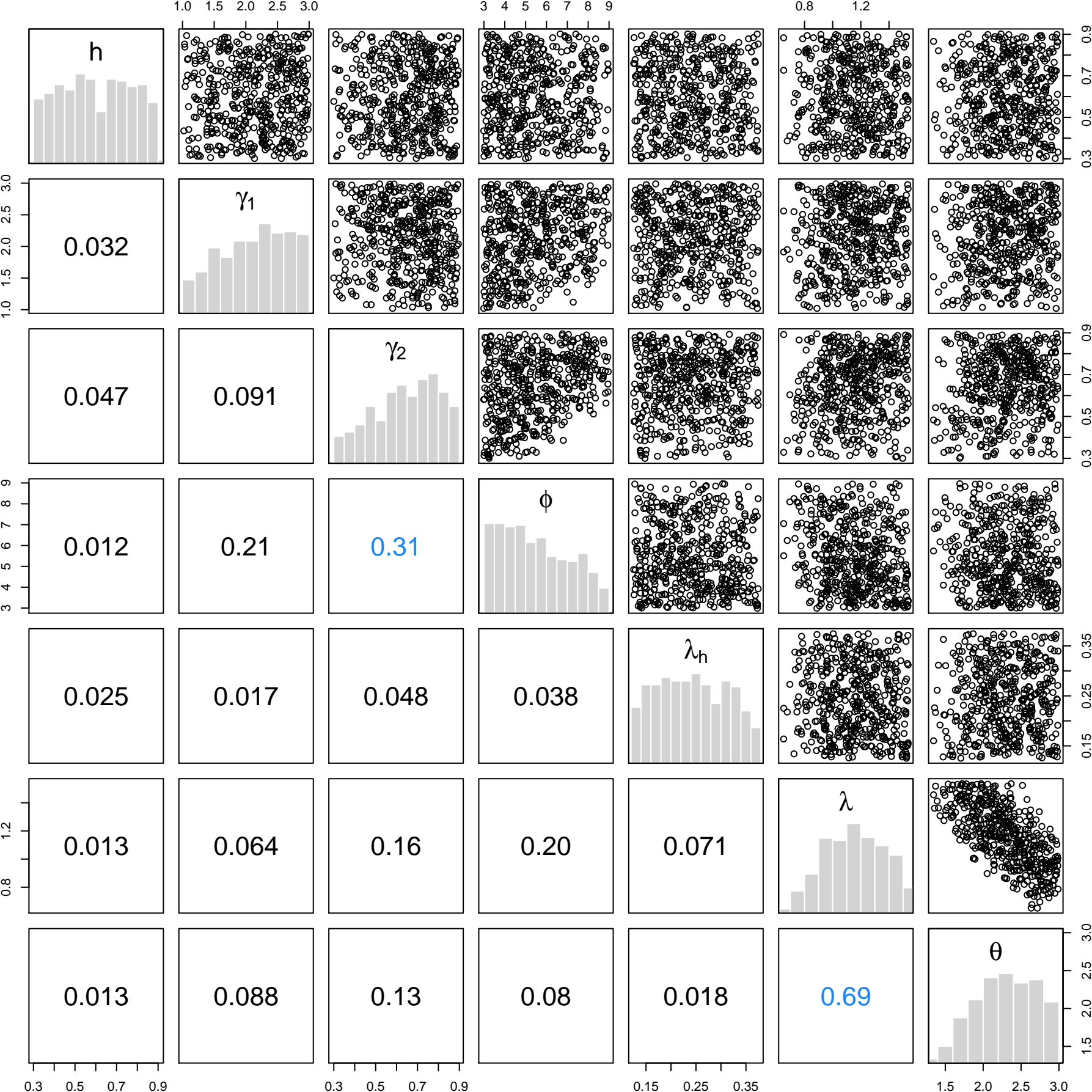

Supplement: S15 Fig — Correlation coefficients (absolute value) are shown in the subdiagonal plots; values greater than 0.25 are highlighted in blue. The underlying data and code to generate this figure may be obtained by running the file “ebola-forecasting-supplement.R” deposited in the Dryad repository: http://doi.org/10.5061/dryad.17m5q. (PDF) [file pbio.1002056.s015.pdf]
