# Supplementary material for: Ebola Cases and Health System Demand in Liberia
Source: PLoS Biol. 2015 Jan 13;13(1):e1002056. doi: 10.1371/journal.pbio.1002056 (PMC4293091; doi:10.1371/journal.pbio.1002056)
Supplement: S11 Fig — The underlying data and code to generate this figure may be obtained by running the file “ebola-forecasting-supplement.R” deposited in the Dryad repository: http://doi.org/10.5061/dryad.17m5q. (PDF) [file pbio.1002056.s011.pdf]

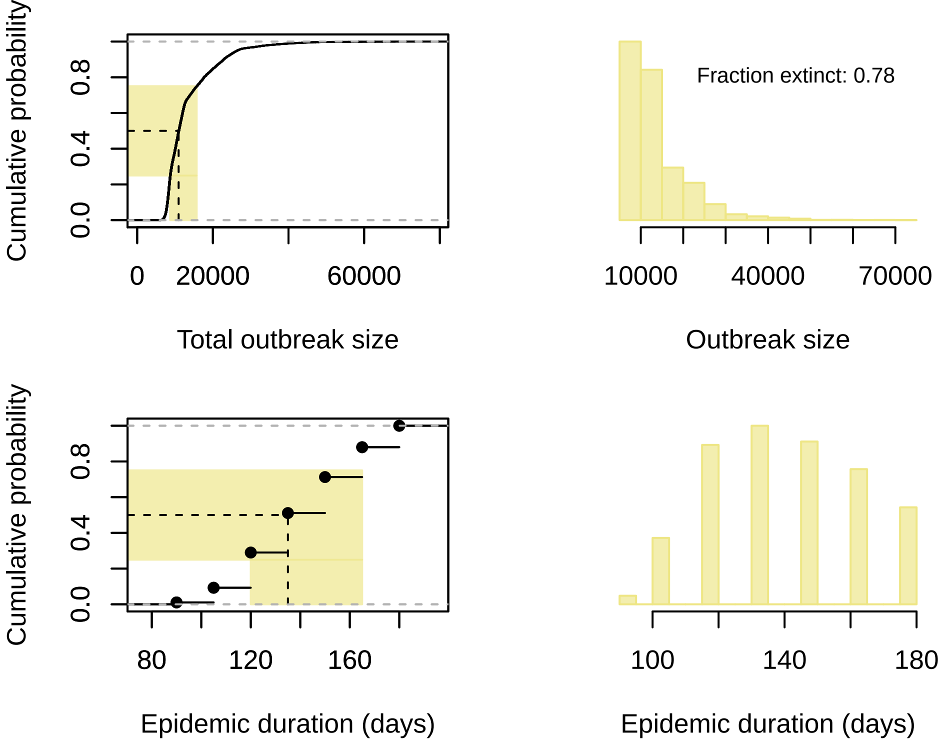
Cumulative probability
0.0
0.4
0.8
0
20000
60000
Total outbreak size
Fraction extinct: 0.78
10000
40000
70000
Outbreak size
Cumulative probability
0.0
0.4
0.8
80
120
160
Epidemic duration (days)
100
140
180
Epidemic duration (days)
