# Supplementary material for: Ebola Cases and Health System Demand in Liberia
Source: PLoS Biol. 2015 Jan 13;13(1):e1002056. doi: 10.1371/journal.pbio.1002056 (PMC4293091; doi:10.1371/journal.pbio.1002056)
Supplement: S1 Text — (PDF) [file pbio.1002056.s016.pdf]

Ebola cases and health system demand in Liberia: Supplementary Text

John M. Drake, RajReni B. Kaul, Laura W. Alexander, Suzanne M. O'Regan, Andrew M. Kramer, J. Tomlin Pulliam, Matthew J. Ferrari, and Andrew W. Park

Introduction

This document contains supplementary material for the paper "Ebola cases and health system demand in Liberia" by the UGA-MIDAS Ebola Modeling Group.

Effective reproduction number (R_{eff})

From the independence of the mixture components, we obtain the *mean matrix* for transmission generations defined in terms of treatment location.

$$M = \begin{bmatrix} Nq\alpha\beta\theta + h\lambda_h & (1-h)\lambda_h \\ h(\theta Nqg + (1-g)(Nq\theta + \phi)) & (1-h)(\theta Nqg + (1-g)(Nq\theta + \phi)) \end{bmatrix} \tag{1}$$

The two eigenvalues of this matrix are:

$$\begin{aligned} \Lambda_1 = &\frac{1}{2}(Nq(1-h+\alpha\beta)\theta + (g-1)(h-1)\phi + h\lambda_h \\ &+ \sqrt{((Nq\phi(h+\alpha\beta-1) - (g-1)(h-1)\phi)^2 + h\lambda_h(2Nq\phi(1-h+\alpha\beta) + 2\phi(g-1)(h-1) + h\lambda_h)))} \end{aligned} \tag{2}$$

$$\begin{aligned} \Lambda_2 = &\frac{1}{2}(Nq(1-h+\alpha\beta)\theta + (g-1)(h-1)\phi + h\lambda_h \\ &- \sqrt{((Nq\phi(h+\alpha\beta-1) - (g-1)(h-1)\phi)^2 + h\lambda_h(2Nq\phi(1-h+\alpha\beta) + 2\phi(g-1)(h-1) + h\lambda_h)))} \end{aligned} \tag{3}$$

The dominant eigenvalue (Λ) is the long run growth rate of the epidemic and provides a threshold criterion such that outbreak will grow if $\Lambda > 1$ and decline if $\Lambda < 1$. In this model, which ignores susceptible depletion, Λ is always the effective reproduction number (R_{eff}) in that it is the average number of secondary infections in a population comprised of community-treated and hospital-treated cases at its stable distribution. If evaluated at $t = 0$, Λ may also be interpreted as the basic reproductive ratio (R_0). A special case of interest is the complete elimination of cases in the community generated by cases treated in the hospital ($\lambda_h = 0$). In this case, the eigenvalues are $\Lambda_1 = \alpha\beta\theta Nq$ and $\Lambda_2 = (1-h)((1-g)(\theta Nq + \phi) + g\theta Nq)$. Which Λ will be dominant depends on the values of α, β, h, g, and ϕ, so that eventually either community transmission or hospital transmission drives the persistence of the infection. Further insight may be obtained by inspecting the case where funeral transmission is reduced to zero ($\phi = 0$). Then, $\Lambda_2 = (1-h)\theta Nq$. Community transmission dominates in this case if $1-h > \alpha\beta$. Note that where hospital transmission dominates ($\Lambda_1 > \Lambda_2$) the elasticities of the parameters are identical. This means that proportional changes in each quantity have identical effect (halving the contact number is equivalent to halving the effectiveness of infection control is equivalent to halving the increased contact rate in health care facilities, *etc.*).

Initial Forecasts

To forecast future cases under different scenarios for aid and intervention in the fall of 2014, we projected cases and number of persons seeking hospitalization from 3 September 2014 until 31 December 2014 (120 days) under five scenarios:

1. *Baseline.* Transmission and hospitalization continue at pre-intervention levels (hospital capacity of 601 beds);
2. *Scenario A.* Conditions improve due to the U.S. aid commitment of 15 September 2014 (hospital capacity increases by 1,700 beds in Ebola treatment centers between 25 October 2014 and 28 December 2014 to a total of 2,301 beds);
3. *Scenario B.* Conditions improve through an increase in hospital capacity of 6,800 new beds (four times the U.S. aid commitment of 1,700 beds), bringing total hospital capacity to 7,401 bed equivalents by 28 December 2014;
4. *Scenario C.* Conditions improve by increase in hospital capacity to 7,401 bed equivalents by 28 December 2014 and hospital admission rate of 85%.
5. *Scenario D.* Conditions improve by increase in hospital capacity to 7,401 bed equivalents by 28 December 2014 and hospital admission rate of 99%.

Initial conditions for these scenarios were derived from outbreak reports issued by the Liberia Ministry of Health and World Health Organization. Specifically, on 2 September 2014 the number of persons per infection generation that could be treated in ETUs was 1444. In this generation, the number of reported infected persons was $1871 - 972 = 899$ for a total infection generation of approximately 2248. We assume dthat, at most, the fraction seeking hospitalization (60.2%) was admitted, yielding $2248 \times 0.602 \approx 1353$ with $2248 - 1353 = 895$ remaining in the community.

Scenario A assumed that the Department of Defense (DoD) improvements to hospital capacity constitute the main intervention against the continued spread of Ebola virus in West Africa. In this scenario, an additional 1,700 hospital beds would have become available between 25 October and 28 December at a rate of one 100-bed facility every four days, based on the expectation that all units would be operational by the end of 2014. Results suggest that an initial downturn in cases was to be expected based on isolation, but that capacity would eventually be outstripped by a secondary rise in cases. While these results do predict a temporary downturn, they do not imply that hospitalization would ave been exclusively responsible for this trend. Particularly, public compliance with burial policies, actions taken to increase personal safety, and deterioration in reporting would also have played a role.

Scenario B assumed that the main line of intervention would be further improvements to hospital capacity in excess of DoD improvements in Scenario A. In this scenario, an additional 6,800 “bed equivalents” (which may include Ebola Community Care units as well as other units) became available between 25 October and 28 December (including the 1,700 ETU beds from Scenario A). The outcome of this scenario is interesting because it shows that improved treatment facilities are not enough to ensure containment (see supporting information). As above, the increased availability of treatment slows transmission for a time, but the outbreak outgrows capacity and takes off again. Although the upper end of the distribution is reduced, the median and lower end are similar to Scenario A, suggesting that hospital capacity was unlikely to be the limiting factor after the DoD improvements are complete.

Scenario C assumed that improved hospital capacity would be complemented by improved public compliance with recommendations. In this scenario, the fraction of infected persons seeking hospitalization was increased from its baseline (a variable number around 60%) to 85% in addition to the increased hospital capacity envisioned in Scenario B. The expected (median) outcome of this scenario was containment, although some initially plausible parameterizations could not be contained by this strategy.

Although the expected (median) solution to Scenario C was rapid containment with new cases peaking in mid-December, this outcome was not guaranteed: there were several plausible parameterizations for which the interventions were inadequate. We therefore investigated a further scenario (Scenario D) designed to achieve

containment. In this scenario, the number of additional bed equivalents was again set to 6,800 and public compliance with hospitalization was 99%. A majority of parameterizations resulted in containment. Since containment was typically achieved by this scenario, we ran the simulation until elimination in a majority of cases.

Supplementary Figures

S1 Fig shows the distribution of five case classifications over the time period used for fitting. These are: (1) number of health care workers infected (HCW), (2) total number of reported cases, (3) total cases, (4) fraction of cases that were hospital-acquired, and (5) fraction of cases associated with funeral preparation and burial. For (1) and (2), the red line corresponds to the WHO reported number during the interval. For (3), the red line corresponds to total cases obtained by multiplying the number of reported cases by 2.5, the presumed factor of under-reporting. Importantly, the number of funeral acquired cases is consistent with anecdotes reported by Rivers et al [17]. The fraction of cases that are hospital-acquired is somewhat lower than anecdotally reported.

S2 Fig projects daily hospital demand (new patients seeking hospitalization) according to the baseline scenario with simulations starting on 2 September 2014.

S3 Fig and S4 Fig illustrate the range of trajectories and daily hospital demand associated with Scenario A.

S5 Fig and S6 Fig illustrate the range of trajectories and daily hospital demand associated with Scenario B.

S7 Fig and S8 Fig illustrate the range of trajectories and daily hospital demand associated with Scenario C.

S9 Fig and S10 Fig illustrate the range of trajectories and daily hospital demand associated with Scenario D.

S11 Fig shows the total epidemic duration and outbreak size for the 78% of simulations of Scenario D terminating in mid summer 2015. S11 Fig also shows the cumulative probability distribution for outbreak size and duration and histograms of outbreak size and duration from the $10,450$ simulations.

Model sensitivity

This study used the novel method of plausible parameter sets to represent model uncertainty. To investigate the sensitivity of this approach to parameters of the fitting process, we performed a number of secondary investigations and sensitivity analyses. The latin hypercube space sampled in the analysis reported in the main text was based on an interval of +/-25% of the least squares fit for each parameter. To investigate the influence of this choice, we re-ran the latin hypercube sampling over a much wider range (+/-50% of the least squares fit). Unsurprisingly, this yielded a smaller fraction of plausible parameterizations (10.0% compared with 20.1%). These parameter sets were then run through the five intervention scenarios (S12 Fig). These simulations show that even very different endpoints to the sampled parameter region do not change the primary conclusions of this study: (i) the expected total number of cases will almost certainly exceed 10,000 regardless of the size of the response, (ii) the most likely epidemic size is between 10,000 and 50,000 cases for all intervention scenarios, and (iii) the effect of intervention is primarily to reduce the chance of a very large epidemic (which is significantly reduced by Scenario A and almost eliminated by Scenario B). Further, this plot shows that the range of potential outcomes identified in the main paper is not greatly affected by increasing the parameter space sampled (the endpoints of the blue regions and gray lines in S12 Fig are roughly coincident), but if we had limited our investigation to the smaller parameter region, we would have overlooked a substantial number of plausible parameter sets yielding intermediate sized epidemics.

The extent to which different parameters may trade off against each other to achieve alternative plausible parameterizations is reflected in the correlations among parameters in parameter sets that have been identified to be consistent with the observed data. S13 Fig shows all parameter pairs in the plausible parameter sets from the latin hypercube sample obtained using +/-25% of the parameters estimated by least squares. Correlation coefficients (absolute value) are shown in the subdiagonal plots; values greater than $\rho = 0.25$ are

indicated in blue. For comparison, S14 Fig and S15 Fig show correlations among parameter pairs from the sample using +/-50% and +/-10% of the estimated parameters, respectively.
